# Use of intranasal mupirocin to prevent methicillin-resistant *Staphylococcus aureus *infection in intensive care units

**DOI:** 10.1186/cc3512

**Published:** 2005-03-31

**Authors:** Arno Muller, Daniel Talon, Alexandre Potier, Evelyne Belle, Gilles Cappelier, Xavier Bertrand

**Affiliations:** 1 Student, Service d'Hygiène hospitalière et d'Epidémiologie moléculaire, Centre Hospitalier Universitaire Jean Minjoz, Besançon, France; 2 Head of Department, Service d'Hygiène hospitalière et d'Epidémiologie moléculaire, Centre Hospitalier Universitaire Jean Minjoz, Besançon, France; 3 House Officer, Service de Réanimation médicale Centre Hospitalier Universitaire Jean Minjoz, Besançon, France; 4 Clinician, Service de Réanimation médicale Centre Hospitalier Universitaire Jean Minjoz, Besançon, France; 5 Head of Department, Service de Réanimation médicale Centre Hospitalier Universitaire Jean Minjoz, Besançon, France; 6 Clincian, Service d'Hygiène hospitalière et d'Epidémiologie moléculaire, Centre Hospitalier Universitaire Jean Minjoz, Besançon, France

## Abstract

**Introduction:**

Methicillin-resistant *Staphylococcus aureus *(MRSA) causes severe morbidity and mortality in intensive care units (ICUs) worldwide. The purpose of this study was to determine whether intranasal mupirocin prophylaxis is useful to prevent ICU-acquired infections with MRSA.

**Materials and methods:**

We conducted a 4-year observational retrospective study in a 15-bed adult medical ICU. During the first 2-year period mupirocin ointment was included in the MRSA control programme; during the second, mupirocin was not used. The main endpoint was the number of endogenous ICU-acquired infections with MRSA.

**Results:**

The number of endogenous acquired infections was significantly higher during the second period than during the first (11 versus 1; *P *= 0.02), although there was no significant difference in the total number of patients infected with MRSA between the two periods. We also observed that nasal MRSA decolonisation was significantly higher in the mupirocin period than in mupirocin-free period (*P *= 0.002).

**Conclusion:**

Our findings suggest that intranasal mupirocin can prevent endogenous acquired MRSA infection in an ICU. Further double-blind, randomised, placebo-controlled studies are needed to demonstrate its cost-effectiveness and its impact on resistance.

## Introduction

Over the past four decades, methicillin-resistant *Staphylococcus aureus *(MRSA) has spread throughout the world and become highly endemic in many geographic areas. This pathogen causes severe morbidity and mortality in hospitals worldwide [1-3]. In France, 30 to 40% of *S. aureus *strains are methicillin-resistant and the median incidence of MRSA in clinical specimens is about 0.8 per 1000 patient-days in acute care facilities and 3.42 in intensive care units (ICUs) [4]. French intensive care experts had recommended that barrier precautions should be implemented for patients colonised or infected with MRSA, but there is no consensus about an MRSA screening programme and MRSA nasal decolonisation. Systematic MRSA screening on admission and preventive isolation have been shown to be cost-effective [5-7] but the usefulness of mupirocin-based nasal decolonisation remains a matter of debate. Indeed, mupirocin has emerged as the topical antibacterial agent of choice for the elimination of *S. aureus *nasal carriage. In hospitals in which MRSA is epidemic, the intra-nasal administration of mupirocin to both patients and personnel colonised with MRSA is considered to be appropriate [8]. Furthermore, mupirocin has been used successfully to prevent staphylococcal infections in surgical and haemodialysis patients [9]. Although the available literature does not support routine use of intranasal mupirocin to prevent subsequent infections, there may be a role in some selected cases, such as those involving critically ill patients [9]. Thus, it was reported that intranasal mupirocin could decrease the occurrence of *S. aureus *pneumonia in ICU patients [10]. In 1994, an MRSA control programme was implemented in all high-risk units of our hospital, including adult ICUs. Thus, all patients were screened for MRSA on admission and during hospitalisation; MRSA-positive patients were kept in isolation and positive patients were prescribed nasal mupirocin ointment. In June 2001, clinicians from the medical ICU decided to stop using mupirocin for MRSA nasal decolonisation. We conducted a retrospective study to determine whether intranasal mupirocin prophylaxis is useful in preventing ICU-acquired infections with MRSA.

## Materials and methods

### Setting and study period

The 15-bed medical ICU of Besançon hospital admits about 450 to 500 patients per year, giving a mean of 5,000 patient-days per year. All patients admitted between 1 June 1999 and 31 May 2003 were included in this retrospective study. Mean ICU length of stay, mean gravity scores (Simplified Acute Physiology Score, SAPSII) and fatality rates were calculated monthly [11]. Ethical approval for this study was granted by the ethical committee of the hospital.

### MRSA control programme

The MRSA control strategy was based on screening nasal fluid samples from all patients for MRSA on admission and once a week during hospitalisation. When the screening test was positive for MRSA, patients were given nasal mupirocin for 5 days, even if other body sites were colonised with MRSA. Special precautions were taken to prevent cross-contamination, including the use of disposable gowns and gloves, the use of an alcohol rub for hand hygiene, and the implementation of strict environmental hygiene measures. The MRSA status of the patient was written on the door of their room and in their medical chart. The patient was removed from isolation when two consecutive screening tests were negative and no clinical samples tested positive for MRSA. The programme did not include restrictions on antibiotic use. This programme was applied in our medical ICU until June 2001. At that date, nasal mupirocin ointment was stopped.

### Microbiological techniques

The screening programme involved the collection of nasal samples from each patient and of tracheal aspirates from ventilated patients. Clinical diagnostic samples were obtained as requested by the physician in charge of the patient. Screening samples were used to inoculate both Mueller–Hinton agar and Mueller–Hinton agar supplemented with 10 mg/l tobramycin because 20% of all MRSA isolated in our hospital are tobramycin-susceptible [12,13]. Plates were examined for staphylococci after 24 hours at 37°C. Identification of *S. aureus *was initially based on colony aspect and the detection of both clumping factor and protein A with the Pastorex Staph-Plus latex agglutination test (Bio-Rad, Marnes la Coquette, France). Complementary tests, such as the coagulase and DNase tests, were performed if necessary. *S. aureus *strains that grew both on plates with and on those without tobramycin were considered to be MRSA. Indeed, a continuous surveillance of antimicrobial resistance among clinical samples showed that more than 98% of methicillin-susceptible *Staphylococcus aureus *were susceptible to tobramycin. Those that grew only on tobramycin-free plates were tested for oxacillin resistance. This antibiotic susceptibility was determined with the disk diffusion technique by incubating with 5 μg oxacillin disks for 48 hours at 30°C, as recommended by the Antibiogram Committee of the French Society for Microbiology [14]. *S. aureus *strains with oxacillin inhibition zones of less than 20 mm (corresponding to a minimum inhibitory concentration of more than 2 mg/l) were classified as MRSA. About 75% of the positive MRSA screening results were obtained within 24 hours; the others (25%) were obtained within 48 hours.

### MRSA definitions

The following definitions were used: case, a patient from whom MRSA was recovered from any site irrespective of the sample type (screening or clinical); carrier, a patient from whom MRSA was recovered from screening samples or from clinical samples without signs of infection; and infected patient, a patient from whom MRSA was recovered from clinical samples with signs of infection according to the definitions of the Centers for Disease Control and Prevention [15]. MRSA was considered to be imported if the patients tested positive within 48 hours of admission in the ICU and to be acquired MRSA if they tested negative for the first 48 hours after admission. Infections were considered to be endogenous if a screening sample was positive before the clinical sample, or exogenous otherwise. MRSA was considered to have been eradicated when two consecutive screening tests were negative.

### Antibiotic use

The quantities of each antimicrobial delivered to the ICU were determined from the pharmacy information system. Grams and international units of antimicrobials were further converted into defined daily doses (DDD) in accordance with the recommendations of the World Health Organization [16]. The amount of each class of antibiotics used is expressed in DDD per 1,000 days of hospitalisation.

### Statistical analysis

The aim of the study was to compare ICU-acquired infections with MRSA before (period 1, June 1999 to May 2001) and after (period 2, June 2001 to May 2003) the use of intranasal mupirocin stopped. Analysis was performed by using the χ^2 ^test and Fisher's exact test for categorical variables and the Mann–Whitney test for continuous variables. *P *≤ 0.05 was considered to be significant. Statistical analysis was performed with Epi-info 6.0 (Centers for Disease Control and Prevention, Atlanta, GA) and R (The R Project for Statistical Computing, ) software.

## Results

During the first study period with mupirocin use, 912 patients were admitted to the medical ICU, 775 were screened for MRSA (84.5%), 38 were MRSA carriers and 9 were infected with MRSA. During the second period without mupirocin use, 987 patients were admitted, 819 were screened (83.0%), 49 were MRSA carriers and 17 were infected with MRSA (Table 1). The number of endogenous acquired infections was significantly higher during the second period than during the first (11 versus 1; *P *= 0.006, relative risk = 8.53 (CI95% [1.15–63.21])) although there was no significant difference in the total number of patients infected with MRSA between the two periods. The 12 endogenous acquired infections detected are described in Table 2. In the second period, the delay between the detection of MRSA carriage and the date of the first positive clinical sample was sufficient to implement nasal mupirocin ointment for 7 of the 11 infected patients. Three patients died during the 2 weeks after the MRSA bacteraemia or pneumonia.

We next evaluated the efficiency of nasal mupirocin for MRSA eradication. The rate of nasal MRSA decolonisation among the patients hospitalised for more than 14 days after the discovery of MRSA carriage (delay necessary to evaluate the efficacy of mupirocin) reached 86.4% in period 1 and just 46.4% in period 2 (*P *= 0.002). Three of the 19 patients who were successfully decolonised during the first period received concomitant treatment with antimicrobials effective against MRSA (namely vancomycin).

The total amount of antibiotics used remained stable during the study period (1,700 DDD per 1,000 hospitalisation days) and the use of glycopeptides did not significantly vary (104 DDD per 1,000 hospitalisation days in the first period and 91 in the second period).

## Discussion

Our observational study suggests that nasal mupirocin can effectively prevent the occurrence of endogenous acquired MRSA infections in ICUs. The increase in endogenous ICU-acquired MRSA infections in the second period occurred although different indicators such as mean length of stay, mean gravity scores of fatality rates did not significantly vary between the periods (Table 1). In 7 of the 11 MRSA infections observed in the period 2, the delay between the two samples was sufficient to implement mupirocin treatment; that is, at least 5 days. Our findings stand in contrast to double-blind randomised, placebo-controlled trials that included patients hospitalised in different type of unit [17]. However, in this study, the number of infections was three in the mupirocin group and seven in the placebo group. It is likely that the enrolment of more than 98 patients would show a significant effect of mupirocin in preventing subsequent MRSA infection. Moreover, the only randomised study applied to ICU patients also concluded that mupirocin was effective in reducing the occurrence of *S. aureus *pneumonia [10].

Some limitations of our study have to be addressed. First, we compared historical groups, whereas randomised, double-blind placebo controlled trials are more powerful. However, MRSA infection remains a rare event in our medical ICU and a very large study period would be needed to show that a programme including mupirocin use is beneficial. We simply reported the evolution of the infections with MRSA between two periods during which only one element had changed: the use of mupirocin for nasal MRSA decolonisation.

Second, we have not evaluated mupirocin resistance in this study. Low-level mupirocin resistance has been identified as a risk factor for a failure to eradicate MRSA [18], and numerous authors have reported the emergence of mupirocin resistance in settings where mupirocin is commonly used [19]. Concerns over the development of resistance have dissuaded many hospitals from using mupirocin in this manner, and recent recommendations from the guidelines of the Society for Healthcare Epidemiology of America stated that 'widespread use, prolonged use or both of decolonization therapy should be avoided' [20]. Others studies have reported that relapse of MRSA carriage was not associated with the development of resistance to mupirocin [21,22].

The body of the literature currently does not support routine intranasal mupirocin prophylaxis to all inpatients to decrease the rate of clinical infection. However, there might be as yet unidentified patient populations that could benefit. One can speculate that a significant effect should be seen for patients at high risk of infection, such as patients admitted to ICUs [23]. Indeed, our data showed that the infectious risk in our ICUs is high; during period 2 (mupirocin-free), 25.6% of carriers became infected (Table 1). A recent meta-analysis reported a significant increase in mortality associated with MRSA infection (odds ratio 1.93) and most studies depicted a crude mortality rate between 20% and 40% [3].

Our results concerning MRSA nasal eradication are in agreement with those observed by several authors in different settings, Kalmeijer and colleagues reported that MRSA was eradicated from 83.5% of patients admitted for orthopaedic surgery [24], and Mody and colleagues also showed that treatment with mupirocin had decolonised 93% of residents in long-term care facilities [21]. However, they differed from those obtained in other randomised trials. This difference was probably due to the status of the patients included, regardless of the type of setting.

## Conclusion

Our results suggest that the use of mupirocin to prevent MRSA infections in ICUs has to be evaluated. Further double-blind, randomised, placebo-controlled clinical trials are needed to demonstrate its cost-effectiveness and its impact on mupirocin resistance.

## Key messages

• 	MRSA causes severe morbidity and mortality in ICUs worldwide.

• 	Mupirocin is regarded as the topical antibacterial agent of choice for the elimination of *S. aureus *nasal carriage.

• 	The body of the literature does not currently support routine intranasal mupirocin prophylaxis.

• 	Our observational study suggests that nasal mupirocin can effectively prevent the occurrence of endogenous acquired MRSA infections in ICUs.

• 	Further double-blind, randomised, placebo-controlled clinical trials are needed to confirm our findings in ICUs.

## Abbreviations

DDD = defined daily doses; ICU = intensive care unit; MRSA = methicillin-resistant *Staphylococcus aureus*.

## Competing interests

The author(s) declare that they have no competing interests.

## Authors' contributions

AM collected the data and performed the statistical analysis. AP recorded the clinical information. XB and DT composed the writing committee. EB and GC supervised AP for the record of clinical information. All authors read and approved the final manuscript.

## Figures and Tables

**Table 1:** Description of the medical intensive care unit of Besançon hospital, June 1999 to May 2003

Parameter	Period 1 (June 1999 to May 2001)	Period 2 (June 2001 to May 2003)	*P*
No. of patients admitted	912	987	
Patients screened for MRSA (% of patients admitted)	775 (84.5%)	819 (83.0%)	0.26
Mean length of stay, days	11.44	9.73	**0.02**
Mean gravity score, SAPS II	46.32	46.44	0.91
Rate of death, %	32.73	28.43	0.07
MRSA cases	46	55	0.59^e^
Carriers of MRSA^a ^(then infected)	38 (1)	49 (11)	0.35^g^
Infected with MRSA^b^	9	17	0.24^e^
Imported MRSA^c^	6	4	0.66^e^
Exogenous acquired MRSA^c,d^	2	2	1.00^f^
Endogenous acquired MRSA^c,d^	1	11	**0.006**^g^

^a^Patients from whom methicillin-resistant *Staphylococcus aureus *(MRSA) was recovered from screening samples or from clinical samples without signs of infection. ^b^Patients from whom MRSA was recovered from clinical samples with signs of infection in accordance with the definitions of the Centers for Disease Control and Prevention. ^c^Defined as imported if they tested positive within 48 hours of admission, and as acquired if they tested negative for the first 48 hours after admission. ^d^Defined as endogenous if a screening sample was positive before the clinical sample, and as exogenous otherwise. ^e^In comparison with admitted patients. ^f^In comparison with screened patients. ^g^In comparison with carriers of MRSA. SAPS II, Simplified Acute Physiology Score, SAPS II.

**Table 2:** Characteristics of endogenous invasive MRSA infections

Patient no.	Type of infection	Period	Date of infection	Delay between admission and infection (days)	Delay between positive MRSA screening and infection (days)	Antibiotic treatment (Y/N)	Death (Y/N)	Time between infection and death (days)
1	Pneumonia	P1	4 Sep 2000	26	7	Y (vancomycin)	N	
2	Bacteraemia	P2	14 Aug 2001	8	7	Y (vancomycin)	Y	15
3	Pneumonia	P2	29 Aug 2001	37	6	Y (vancomycin)	N	
4	Pneumonia	P2	29 Aug 2001	13	2	N	Y	0
5	Endocarditis	P2	10 Oct 2001	6	6	Y (vancomycin)	N	
6	Bacteraemia	P2	24 Dec 2001	18	13	Y (vancomycin)	N	
7	Bacteraemia	P2	16 Feb 2002	8	5	Y (vancomycin)	N	
8	Pneumonia	P2	1 Apr 2002	3	2	Y (vancomycin)	N	
9	Pneumonia	P2	6 Jun 2002	13	13	Y (vancomycin)	N	
10	Bacteraemia	P2	22 Jun 2002	26	26	Y (vancomycin)	N	
11	Pneumonia	P2	6 Feb 2003	13	3	Y (linezolid)	Y	7
12	Pneumonia	P2	24 Mar 2003	7	5	Y (vancomycin)	N	

MRSA, methicillin-resistant *Staphylococcus aureus*; P1, June 1999 to May 2001; P2, June 2001 to May 2003.
